# Laparoscopic versus open gastrectomy for locally advanced gastric cancer after neoadjuvant chemotherapy: a comprehensive contrastive analysis with propensity score matching

**DOI:** 10.1186/s12957-023-03221-4

**Published:** 2023-11-09

**Authors:** Chenggang Zhang, Peng Zhang, Jiaxian Yu, Qi Jiang, Qian Shen, Gan Mao, Abu Bakarr Kargbo, Weizhen Liu, Xiangyu Zeng, Yuping Yin, Kaixiong Tao

**Affiliations:** 1 Department of Gastrointestinal Surgery, Union Hospital, Tongji Medical College, Huazhong University of Science and Technology, Wuhan, 430022 Hubei China; 2 Department of General Surgery, Peking Union Medical College Hospital, Chinese Academy of Medical Sciences & Peking Union Medical College, Beijing, China

**Keywords:** Gastric cancer, Laparoscopic surgery, Open surgery, Surgical oncology, Neoadjuvant chemotherapy

## Abstract

**Background:**

Laparoscopic gastrectomy (LG) is increasingly applied in locally advanced gastric cancer (LAGC) after neoadjuvant chemotherapy (NC). However, there is no study to comprehensively evaluate the clinicopathological, prognostic, and laboratory data such as nutrition, immune, inflammation-associated indexes, and tumor markers between LG and open gastrectomy (OG) for LAGC following NC.

**Methods:**

The clinicopathological, prognostic, and laboratory data of LAGC patients with clinical stage of cT2-4aN1-3M0 who underwent gastrectomy after NC were retrospectively collected. The effects of LG and OG were compared after propensity score matching (PSM).

**Results:**

This study enrolled 148 cases, of which 110 cases were included after PSM. The LG group had a shorter length of incision (*P* < 0.001) and was superior to OG group in terms of blood loss (*P* < 0.001), postoperative first flatus time (*P* < 0.001), and postoperative first liquid diet time (*P* = 0.004). No significant difference was found in postoperative complications (*P* = 0.482). Laboratory results showed that LG group had less reduced red blood cells (*P* = 0.039), hemoglobin (*P* = 0.018), prealbumin (*P* = 0.010) in 3 days after surgery, and less reduced albumin in 1 day (*P* = 0.029), 3 days (*P* = 0.015), and 7 days (*P* = 0.035) after surgery than the OG group. The systemic immune-inflammation index and systemic inflammatory response index were not significantly different between the two groups. As for oncological outcomes, there were no significant differences in postoperative tumor markers of CEA (*P* = 0.791), CA199 (*P* = 0.499), and CA724 (*P* = 0.378). The 5-year relapse-free survival rates (*P* = 0.446) were 46.9% and 43.3% in the LG and OG groups, with the 5-year overall survival rates (*P* = 0.742) being 46.7% and 52.1%, respectively; the differences were not statistically significant. Multivariate Cox regression analysis revealed that tumor size ≥ 4 cm (*P* = 0.021) and the absence of postoperative adjuvant chemotherapy (*P* = 0.012) were independent risk factors for overall survival.

**Conclusions:**

LG has faster gastrointestinal recovery, better postoperative nutritional status, and comparable oncological outcomes than OG, which can serve as an alternative surgical method for LAGC patients after NC.

## Background

Gastric cancer is the fifth most common cancer and the fourth leading cause of cancer-related mortality worldwide [1]. In China, approximately 70% of gastric cancer patients are at an advanced stage of cancer when their diagnoses are confirmed. The primary treatment for locally advanced gastric cancer (LAGC) is complete surgical resection with D2 lymph node dissection. Laparoscopic surgery has been widely performed for gastric cancer since the first reported case of laparoscopic distal gastrectomy [2]. The results of the CLASS-01 [3–5] and KLASS-02 trial [6, 7] showed that laparoscopic surgery for distal gastric cancer was safe and feasible; therefore, the 2021 National Comprehensive Cancer Network guidelines recommended laparoscopic surgery for LAGC.

The MAGIC trial indicated that perioperative chemotherapy with epirubicin, cisplatin, and infused fluorouracil for operable adenocarcinomas of stomach or lower esophageal could decrease tumor stage and significantly improve the 5-year progression-free survival and 5-year overall survival (OS) [8]. The EORTC40954 [9], FNCLCC FFCD [10], and RESOLVE [11] trials have successively confirmed the safety and efficacy of neoadjuvant chemotherapy (NC) for LAGC. However, NC can cause tissue fibrosis and edema, which may influence surgical procedures and tissue healing. Li et al. conducted a randomized controlled trial (RCT) confirming the short-term effects of laparoscopic distal gastrectomy for LAGC after NC [12]; however, long-term outcomes are ambiguous because of limited evidence. And most retrospective studies have differences in baseline data. In addition, there is no study to synthetically compare the laboratory indexes such as nutritional status, immune-inflammation conditions, and tumor markers between the two surgical methods, which is of great significance to comprehensively reflect the influence of different surgical methods on patients. Therefore, after balancing the difference in baseline data by propensity score matching (PSM), we comprehensively compared the clinicopathological, laboratory, and prognostic data of the two groups, so as to confirm the non-inferiority of laparoscopic gastrectomy (LG) to open gastrectomy (OG) for LAGC patients following NC.

## Methods

### Patient selection

**Fig. 1:**
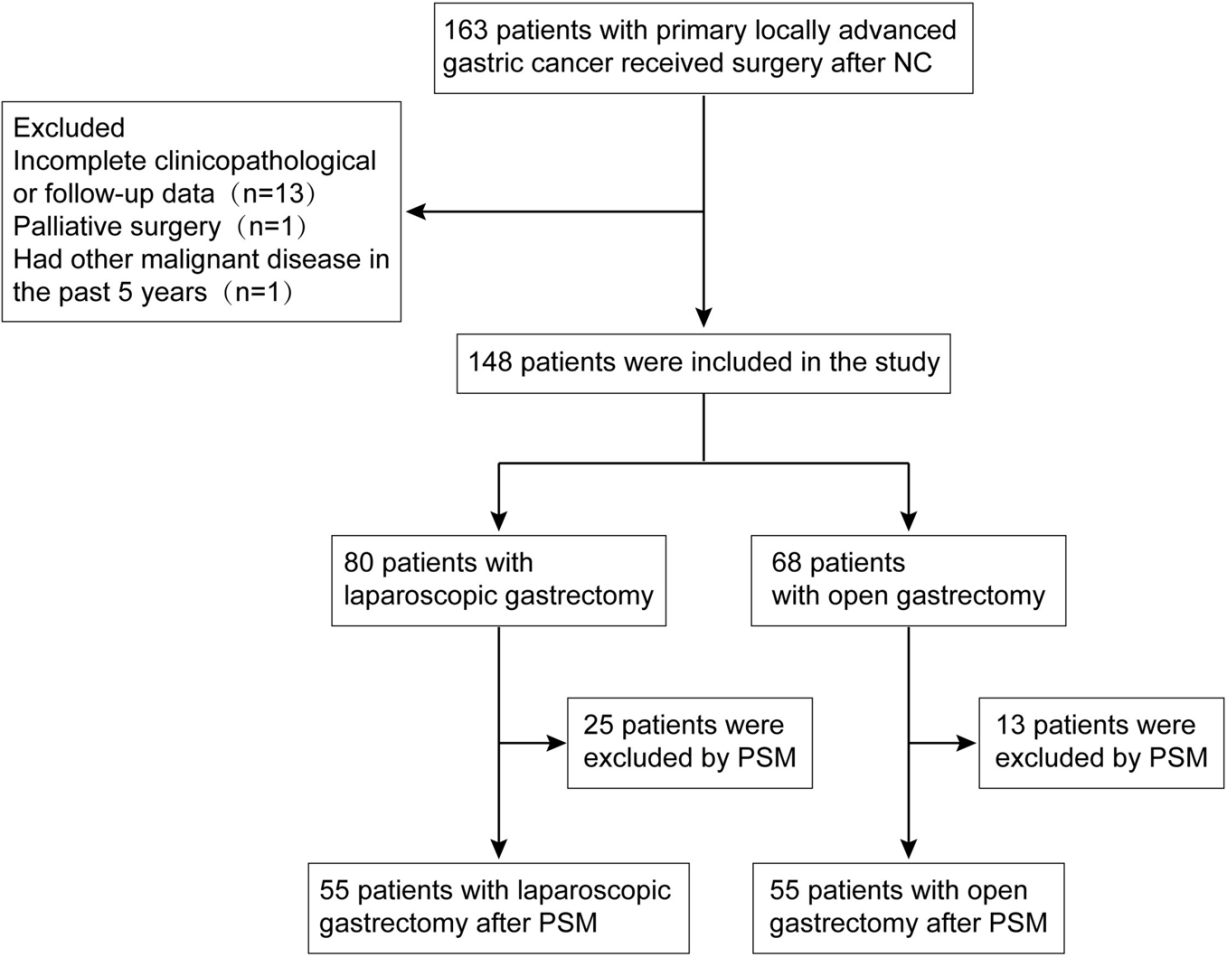
Patients selection flowchart. NC, neoadjuvant chemotherapy; PSM, propensity score matching

This study included patients with LAGC who received surgery after NC between January 2012 and September 2020 in the Department of Gastrointestinal Surgery, Union Hospital, Tongji Medical College, Huazhong University of Science and Technology. The first OG and LG for LAGC patients after NC were conducted at January 2012 and August 2012, respectively. NC was conducted on LAGC patients with a clinical tumor stage of cT2-4aN1-3M0. The treatment regimens included FOLFOX (oxaliplatin, leucovorin, and 5-fluorouracil), SOX (oxaliplatin and TS-1), XELOX (oxaliplatin and capecitabine), and FLOT (docetaxel, oxaliplatin, leucovorin and fluorouracil), which were selected based on the Chinese Society of Clinical Oncology (CSCO) clinical guidelines of gastric cancer [13] and the tolerance of patients. The following were the criteria for inclusion: (1) clinical tumor stage of cT2-4aN1-3M0, (2) underwent surgery after NC. And the following were the criteria for exclusion: (1) palliative gastrectomy, (2) diagnosis of any other malignant tumor in the past 5 years and (3) incomplete clinicopathological or follow-up data. All patients were separated into two groups: those who received LG (LG group) and those who underwent OG (OG group) after NC. The flow chart for the selection of cases is shown in Fig. 1. The Tongji Medical College Ethics Committee of Huazhong University of Science and Technology ratified the protocol of this study and the approved number is UHCT-IEC-SOP-016–03-01. The informed consent was signed by all patients to have their data used for the study.

### Surgical procedure

All surgeries were performed by experienced surgeons of the Department of Gastrointestinal Surgery, Union Hospital, Tongji Medical College, Huazhong University of Science and Technology. Based on previous literatures [4, 7, 12], and physician experience, LG is only considered for patients who meet the following conditions: (1) without abdominal operation before, (2) patients could tolerate laparoscopic surgery, and (3) without obvious metastasis and R0 resection is possible. The criteria for OG are as follows: (1) patients could tolerate surgery, and (2) without obvious metastasis and R0 resection is possible. For patients according to the above criteria, the benefits and risks of the two surgical methods would be fully explained by the surgical physician, and the final decision was made by the patients. The performance of proximal gastrectomy, distal gastrectomy, or total gastrectomy was depended on the tumor location. All patients underwent standard gastrectomy with D2 lymphadenectomy and standard reconstruction based on the Japanese gastric cancer treatment guidelines (5th edition) [14]. Reconstruction after proximal gastrectomy was performed via esophagogastrostomy, while reconstruction after total gastrectomy was performed via Roux-en-Y esophagojejunostomy. Distal gastrectomy reconstruction was performed via standard Roux-en-Y gastrojejunostomy or Billroth II gastrojejunal anastomosis depending on the surgeon’s preference. All reconstruction processes were performed in an open manner. Postoperative adjuvant chemotherapy was routinely applied to all patients unless they could not put up with it due to serious adverse effects. The postoperative adjuvant chemotherapy regimens included XELOX, SOX, FOLFOX, and S-1. For patients who achieved R0 resection after NC, postoperative adjuvant chemotherapy was carried out consistent with the original regimens if NC was effective. If the disease progressed after NC, subsequent chemotherapy regimens would be discussed through multidisciplinary team. Postoperative adjuvant chemotherapy and NC were 8 cycles in total and were adjusted according to the patient’s disease condition and tolerance.

### Data collection and outcome assessment

Demographic data, involving age, sex, body mass index, Charlson Comorbidity Index (CCI) [15], and American Society of Anesthesiologists (ASA) score, were collected from all patients. Enhanced abdominal computed tomography (CT) was performed before NC to evaluate the clinical tumor stage and after NC to assess the chemotherapy response. The *American Joint Committee on Cancer (AJCC) Cancer Staging Manual* (8th) [16] was used to define the clinical tumor stage, and the Response Evaluation Criteria in Solid Tumors (RECIST version 1.1) was applied to evaluate the chemotherapy response [17].

A standard clinical pathway was used for management of all patients. Liquid diet was started after first flatus, and patients were discharged after the absence of complications. Data related to surgery, including the length of incision, operative time, estimated blood loss, time to first flatus, time to first liquid diet, postoperative complications, and length of postoperative hospital stay, as well as pathological characters, were collected. The Clavien–Dindo classification system was applied to grade postoperative complications, which were defined as incidents happening within 30 days after surgery [18, 19]. Tumor regression grade was evaluated according to AJCC standard established by College of American Pathologists (CAP) [20], and pathological stage was defined according to the *AJCC Cancer Staging Manual* (8th) [16]. A 30-day mortality was defined as death due to any cause that occurred within 30 days after surgery.

Laboratory data such as hemocytes, hemoglobin (Hb), total protein (TP), albumin (Alb), and prealbumin (PAB) at 1 day before surgery and 1 day, 3 days, and 7 days after surgery were collected. To avoid the potential influence of inconsistent preoperative baseline levels, the reduced value was calculated as preoperative value subtracted postoperative value to evaluate the outcomes of different surgical methods. The systemic immune-inflammation index (SII) and systemic inflammatory response index (SIRI) were adopted to assess the immune and inflammation conditions at 1 day before surgery and 1 day, 3 days, and 7 days after surgery. The SII was calculated as neutrophil count × platelet count/lymphocyte count. The SIRI was calculated as neutrophil count × monocyte count/lymphocyte count. Tumor markers including CEA, CA199, and CA724 at 1 week before surgery and 1 month after surgery were collected to evaluate the short-term oncological outcomes of two surgical methods.

### Follow-up

Postoperative follow-up was performed every 3–6 months for the first 2 years and every 6–12 months thereafter via outpatient clinics or telephonic interviews. During follow-up, patients’ complete blood count, liver and kidney function, and tumor markers were examined. Chest-, abdomen-, and pelvis-enhanced CT were examined once every 6 months. Gastroscopy was conducted once every year. The last follow-up date was December 30, 2022. After excluding the cases with incomplete clinicopathological or follow-up data, all 148 cases included in this study achieved complete follow-up, and the median follow-up period was 48.0 (3.0–128.8) months. Median follow-up period in LG group was 44.0 (3.2–119.0) months and was 50.0 (3.0–128.8) months in OG group. Relapse-free survival (RFS) was defined as the duration from surgery to recurrence, and OS was defined as the duration from surgery to death. Local recurrence refers to the recurrence at the original surgical site, while distant metastasis refers to the metastasis of organs or parts other than the original surgical site, such as the liver, peritoneum, lung, and bone.

### Propensity score matching

PSM was conducted by matching patients who underwent LG after NC with those who underwent OG after NC to eliminate differences in baseline statistics. Seven covariates (age, sex, CCI, ASA score, clinical stage, tumor size, and resection type) were chosen to calculate the propensity score. PSM was conducted by one-to-one nearest neighbor method, and the caliper was set on 0.1.

### Statistical analysis

Quantitative variables were presented as mean ± standard deviation, and the differences were compared with independent *t*-test. Quantitative variables with high deviation were expressed as medians (interquartile range), and the differences were compared with Mann–Whitney *U*-test. Categorical variables were expressed as frequencies with the differences compared by chi-square test or Fisher’s exact test. Standardized difference was calculated to compare the balance of baseline data before and after PSM. Kaplan–Meier analysis was used to generate the survival curves, and the differences were evaluated by log-rank test. Univariate and multivariate analyses of risk factors for RFS and OS were conducted by the Cox proportional hazards regression model. The cutoff point of the continuous variables associated with survival was determined using the median. The multivariate Cox proportional hazards regression model included variables with *P* < 0.1 in the univariate analysis. Statistical analysis was conducted by SPSS software (SPSS 20.0, Chicago, IL, USA). R (version 4.0.4) was used for PSM. GraphPad Prism software (version 8.0, USA) was used for image processing. *P* < 0.05 was considered statistically significant.

## Results

### Demographic and clinicopathological characteristics

As shown in Fig. 1, 148 patients who underwent gastrectomy after NC were enrolled in the study based on inclusion and exclusion criteria. The LG and OG groups consisted of 80 and 68 participants, respectively. The resection type and anastomotic methods were significantly different before matching. After PSM, 55 patients remained in each group, and the baseline data were comparable between the two groups (Table 1). The LG group included 45 men (81.8%) and 10 women (18.2%) with a mean age of 56.9 ± 9.2 years. The OG group included 46 men (83.6%) and 9 women (16.4%) with a mean age of 57.4 ± 10.8 years.

**Table 1:** Demographic and clinical characteristics in the LG and OG groups before and after PSM

Characteristics	Before PSM			After PSM		
	LG group [*n* = 80 (%)]	OG group [*n* = 68 (%)]	Standardized difference	LG group [*n* = 55 (%)]	OG group [*n* = 55 (%)]	Standardized difference
Age (year)	58.2 ± 8.6	57.4 ± 10.3	0.088	56.9 ± 9.2	57.4 ± 10.8	0.049
Sex						
Male	63 (78.8)	59 (86.8)	0.213	45 (81.8)	46 (83.6)	0.048
Female	17 (21.2)	9 (13.2)	0.213	10 (18.2)	9 (16.4)	0.048
BMI (kg/m^2^)	22.8 ± 3.5	22.9 ± 2.6	0.006	22.9 ± 3.7	22.7 ± 2.2	0.056
CCI^a^						
0	46 (57.5)	49 (72.1)	0.309	40 (72.7)	38 (69.1)	0.079
1	16 (20.0)	13 (19.1)	0.023	8 (14.5)	11 (20.0)	0.146
2	10 (12.5)	3 (4.4)	0.294	4 (7.3)	3 (5.5)	0.074
≥ 3	8 (10.0)	3 (4.4)	0.218	3 (5.5)	3 (5.5)	0
ASA score						
1	10 (12.5)	9 (13.2)	0.021	6 (10.9)	9 (16.4)	0.161
2	69 (86.3)	54 (79.4)	0.184	48 (87.3)	44 (80.0)	0.198
3	1 (1.3)	5 (7.4)	0.302	1 (1.8)	2 (3.6)	0.111
cT stage						
2	2 (2.5)	2 (2.9)	0.025	1 (1.8)	1 (1.8)	0
3	33 (41.3)	30 (44.1)	0.057	23 (41.8)	24 (43.6)	0.036
4a	45 (56.3)	36 (52.9)	0.068	31 (56.4)	30 (54.5)	0.038
cN stage						
0	8 (10.0)	7 (10.3)	0.010	7 (12.7)	7 (12.7)	0
N +	72 (90.0)	61 (89.7)	0.010	48 (87.3)	48 (87.3)	0
cTNM stage^b^						
II	9 (11.3)	8 (11.8)	0.016	7 (12.7)	8 (14.5)	0.053
III	71 (88.8)	60 (88.2)	0.016	48 (87.3)	47 (85.5)	0.053
Tumor location						
Upper third	49 (61.3)	45 (66.2)	0.102	35 (63.6)	36 (65.5)	0.040
Middle third	9 (11.3)	10 (14.7)	0.101	7 (12.7)	8 (14.5)	0.053
Lower third	22 (27.5)	13 (19.1)	0.200	13 (23.6)	11 (20.0)	0.087
Tumor size^c^ (cm)	4.3 ± 2.8	4.2 ± 2.6	0.046	4.2 ± 2.7	4.3 ± 2.7	0.030
Resection type						
Proximal	9 (11.3)	21 (30.9)	0.495	9 (16.4)	11 (20.0)	0.093
Distal	12 (15.0)	9 (13.2)	0.052	8 (14.5)	7 (12.7)	0.053
Total	59 (73.8)	38 (55.9)	0.382	38 (69.1)	37 (67.3)	0.039
Reconstruction method						
Esophagogastrostomy	9 (11.3)	21 (30.9)	0.495	9 (16.4)	11 (20.0)	0.093
Billroth-II gastrojejunostomy	6 (7.5)	4 (5.9)	0.064	4 (7.3)	2 (3.6)	0.164
Roux-en-Y	65 (81.3)	43 (63.2)	0.413	42 (76.4)	42 (76.4)	0

*PSM* propensity score matching, *LG* laparoscopic gastrectomy, *OG* open gastrectomy, *BMI* body mass index, *CCI* Charlson Comorbidity Index, *ASA* American Society of Anesthesiologists

^a^According to the Charlson Comorbidity Index

^b^According to the 8th edition of the *American Joint Committee on Cancer Cancer Staging Manual*

^c^Refer to the long diameter of tumor after neoadjuvant chemotherapy

As shown in Table 2, the most common NC regimens were FOLFOX and SOX in both of the groups. According to the RECIST criteria, 3 patients (2.7%) achieved complete response (CR), 48 patients (43.6%) achieved partial response (PR), 55 patients (50.0%) had stable disease (SD), and 4 patients (3.6) had progressed disease (PD) after NC. Grade 3/4 adverse events of chemotherapy happened in 9.1% (10/110) of patients during the NC period based on the National Cancer Institute Common Terminology Criteria for Adverse Events (CTCAE, version 4.0) [21]. The most common adverse events were neutropenia and myelosuppression. No NC-related death was observed. The postoperative adjuvant chemotherapy portion of the LG group and OG group was 81.8% (45/55) and 83.6% (46/55), respectively, and the difference was not statistically significant (*P* = 0.801).

**Table 2:** Neoadjuvant chemotherapy and pathological characteristics in the LG and OG groups before and after PSM

Characteristics	Before PSM				After PSM			
	LG group [*n* = 80 (%)]	OG group [*n* = 68 (%)]	*Χ*^2^	*p*-value	LG group [*n* = 55 (%)]	OG group [*n* = 55 (%)]	*Χ*^2^	*p*-value
NC regimen			−	0.629			−	0.285
FOLFOX	41 (51.3)	40 (58.8)			25 (45.5)	35 (63.6)		
SOX	31 (38.8)	24 (35.3)			25 (45.5)	17 (30.9)		
XELOX	4 (5.0)	3 (4.4)			2 (3.6)	2 (3.6)		
FLOT	4 (5.0)	1 (1.5)			3 (5.5)	1 (1.8)		
Cycle of NC			3.611	0.164			2.334	0.311
≤ 2	34 (42.5)	39 (57.4)			25 (45.5)	33 (60.0)		
3	22 (27.5)	16 (23.5)			15 (27.3)	11 (20.0)		
≥ 4	24 (30.0)	13 (19.1)			15 (27.3)	11 (20.0)		
Clinical response of RECIST^a^			−	0.748			−	0.504
CR	2 (2.5)	1 (1.5)			2 (3.6)	1 (1.8)		
PR	35 (43.8)	25 (36.8)			27 (49.1)	21 (38.2)		
SD	38 (47.5)	38 (55.9)			25 (45.5)	30 (54.5)		
PD	5 (6.3)	4 (5.9)			1 (1.8)	3 (5.5)		
Tumor regression grade^b^			−	0.357			−	0.264
0	4 (5.0)	1 (1.5)			4 (7.3)	1 (1.8)		
1	6 (7.5)	9 (13.2)			5 (9.1)	8 (14.5)		
2	26 (32.5)	17 (25)			20 (36.4)	14 (25.5)		
3	44 (55.0)	41 (60.3)			26 (47.3)	32 (58.2)		
ypT stage^c^			−	0.221			−	0.266
0	4 (5.0)	1 (1.5)			4 (7.3)	1 (1.8)		
1	6 (7.5)	9 (13.2)			4 (7.3)	8 (14.5)		
2	9 (11.3)	7 (10.3)			7 (12.7)	6 (10.9)		
3	31 (38.8)	36 (52.9)			20 (36.4)	28 (50.9)		
4a	26 (32.5)	13 (19.1)			17 (30.9)	10 (18.2)		
4b	4 (5.0)	2 (2.9)			3 (5.5)	2 (3.6)		
ypN stage^c^			2.679	0.444			3.728	0.292
0	27 (33.8)	18 (26.5)			21 (38.2)	17 (30.9)		
1	14 (17.5)	12 (17.6)			10 (18.2)	9 (16.4)		
2	19 (23.8)	13 (19.1)			12 (21.8)	8 (14.5)		
3	20 (25.0)	25 (36.8)			12 (21.8)	21 (38.2)		
ypTNM stage^c^			−	0.876			−	0.478
PCR	4 (5.0)	1 (1.5)			4 (7.3)	1 (1.8)		
I	10 (12.5)	9 (13.2)			9 (16.4)	9 (16.4)		
II	22 (27.5)	19 (27.9)			14 (25.5)	16 (29.1)		
III	39 (48.8)	34 (50.0)			27 (49.1)	25 (45.5)		
IV	5 (6.3)	5 (7.4)			1 (1.8)	4 (7.3)		
Histological type			0.225	0.635			0.170	0.680
Differentiated	23 (28.8)	22 (32.4)			16 (29.1)	18 (32.7)		
Undifferentiated	57 (71.3)	46 (67.6)			39 (70.9)	37 (67.3)		
Lymph-vascular invasion			1.075	0.300			0.176	0.675
Yes	50 (62.5)	48 (70.6)			38 (69.1)	40 (72.7)		
No	30 (37.5)	20 (29.4)			17 (30.9)	15 (27.3)		
Nerve invasion			0.979	0.322			1.803	0.179
Yes	43 (53.8)	31 (45.6)			34 (61.8)	27 (49.1)		
No	37 (46.3)	37 (54.4)			21 (38.2)	28 (50.9)		
Postoperative adjuvant chemotherapy			0.103	0.748			0.064	0.801
Yes	63 (78.8)	55 (80.9)			45 (81.8)	46 (83.6)		
No	17 (21.3)	13 (19.1)			10 (18.2)	9 (16.4)		

*PSM* propensity score matching, *LG* laparoscopic gastrectomy, *OG* open gastrectomy, *NC* neoadjuvant chemotherapy, *FOLFOX* oxaliplatin, leucovorin, and 5-fluorouracil, *SOX* oxaliplatin and TS-1, *XELOX* oxaliplatin and capecitabine, *FLOT* docetaxel, oxaliplatin, leucovorin, and fluorouracil, *RECIST* Response Evaluation Criteria in Solid Tumors, *CR* complete response, *PR* partial response, *SD* stable disease, *PD* progressed disease, *PCR* pathological complete response

^a^According to the Response Evaluation Criteria in Solid Tumors (RECIST version 1.1)

^b^According to the AJCC standard

^c^According to the 8th edition of the *American Joint Committee on Cancer Cancer Staging Manual*

### The short-term surgical outcomes

The variables related to the short-term surgical outcomes are shown in Table 3. Compared with the OG group, the LG group had a shorter length of incision (6.4 ± 3.2 cm *vs.* 19.1 ± 4.5 cm, *P* < 0.001) and less blood loss (121.7 ± 71.5 ml *vs.* 180.0 ± 89.4 ml, *P* < 0.001), but the operative time was longer (282.0 ± 64.5 min *vs.* 254.6 ± 74.2 min, *P* = 0.041). The proportion of R0 resection (90.9% *vs.* 90.9%, *P* = 1.000) and number of lymph node dissection (LND) (22.5 ± 6.0 *vs.* 24.5 ± 9.2, *P* = 0.180) were not significantly different between the two groups. As for postoperative recovery, it was shown that the LG group had a shorter time to first flatus (3.4 ± 1.2 days *vs.* 4.5 ± 1.8 days, *P* < 0.001) and shorter time to first liquid diet (4.3 ± 1.8 days *vs.* 5.4 ± 2.0 days, *P* = 0.004) than those of the OG group, but there was no significant difference in the median postoperative hospital stay (10.0 days *vs.* 11.0 days, *P* = 0.291).

**Table 3:** Surgical and postoperative short-term outcomes in the LG and OG groups after PSM

Characteristics	LG group (*n* = 55)	OG group (*n* = 55)	*p*-value
Length of incision (cm)	6.4 ± 3.2	19.1 ± 4.5	< 0.001
Operative time (min)	282.0 ± 64.5	254.6 ± 74.2	0.041
Estimated blood loss (ml)	121.7 ± 71.5	180.0 ± 89.4	0.002
Margin of resection			1.000
R0	50	50	
R1	5	5	
Number of lymph node dissection	22.5 ± 6.0	24.5 ± 9.2	0.180
Time to first flatus (day)	3.4 ± 1.2	4.5 ± 1.8	< 0.001
Time to first liquid diet (day)	4.3 ± 1.8	5.4 ± 2.0	0.004
Postoperative hospital stay^a^ (day)	10.0 (9.0, 12.0)	11.0 (9.0, 12.0)	0.291

*PSM* propensity score matching, *LG* laparoscopic gastrectomy, *OG* open gastrectomy

^a^Values are presented as median (*IQR*, interquartile range)

The overall incidence of postoperative complications was 20.9% (23/110), with 10 patients (18.2%) in the LG group and 13 patients (23.6%) in the OG group; the difference was not statistically significant (*P* = 0.482) (Table 4). Grade III or higher postoperative complications occurred in 7 patients (6.4%), including intraperitoneal hemorrhage in 1 patient (OG), delayed gastric emptying in 1 patient (LG), duodenal stump fistula in 1 patient (LG), anastomotic stenosis in 1 patient (OG), respiratory failure in 2 patients (both OG), and cerebral embolism in 1 patient (OG). All these complications were managed by conservative treatment or reoperation as appropriate. No 30-day mortality was recorded.

**Table 4:** Postoperative complications in the LG and OG groups after PSM

Characteristics	LG group [*n* = 55 (%)]	OG group [*n* = 55 (%)]	*Χ*^2^	*p*-value
Overall complications^a^	10 (18.2)	13 (23.6)	0.495	0.482
Grade I	0 (0.0)	1 (1.8)	−	1.000
Delayed wound healing	0 (0.0)	1 (1.8)		
Grade II	8 (14.5)	7 (12.7)	0.077	0.781
Pleural effusion	3 (5.5)	0(0.0)		
Pulmonary infection	1 (1.8)	7 (12.7)		
Intestinal obstruction	3 (5.5)	0 (0.0)		
Wound infection	1 (1.8)	0 (0.0)		
Grade III	2 (3.6)	2 (3.6)	−	1.000
Intraperitoneal hemorrhage	0 (0.0)	1 (1.8)		
Delayed gastric emptying	1 (1.8)	0 (0.0)		
Duodenal stump fistula	1 (1.8)	0 (0.0)		
Anastomotic stenosis	0 (0.0)	1 (1.8)		
Grade IV	0 (0.0)	3 (5.5)	−	0.243
Respiratory failure	0 (0.0)	2 (3.6)		
Cerebral embolism	0 (0.0)	1 (1.8)		

*PSM* propensity score matching, *LG* laparoscopic gastrectomy, *OG* open gastrectomy

^a^According to the Clavien-Dindo classification system

### Perioperative nutritional and immune-inflammation status

**Fig. 2:**
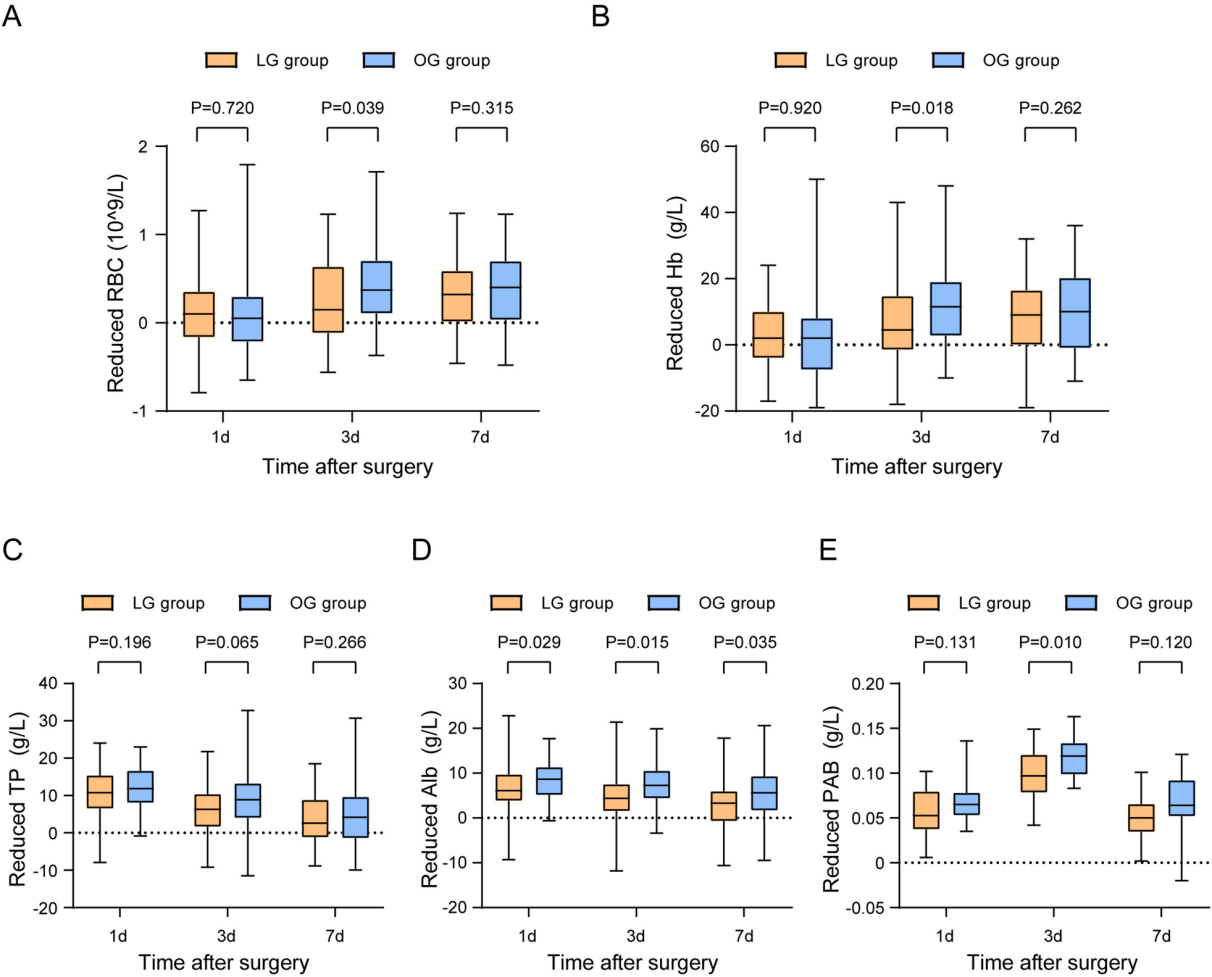
Perioperative nutritional indexes changes in the LG and OG groups. Reduced value was calculated as the value at 1 day before surgery subtracted that of 1 day, 3 days, or 7 days after surgery. LG, laparoscopic gastrectomy; OG, open gastrectomy; RBC, red blood cell; Hb, hemoglobin; TP, total protein; Alb, albumin; PAB, prealbumin

**Fig. 3:**
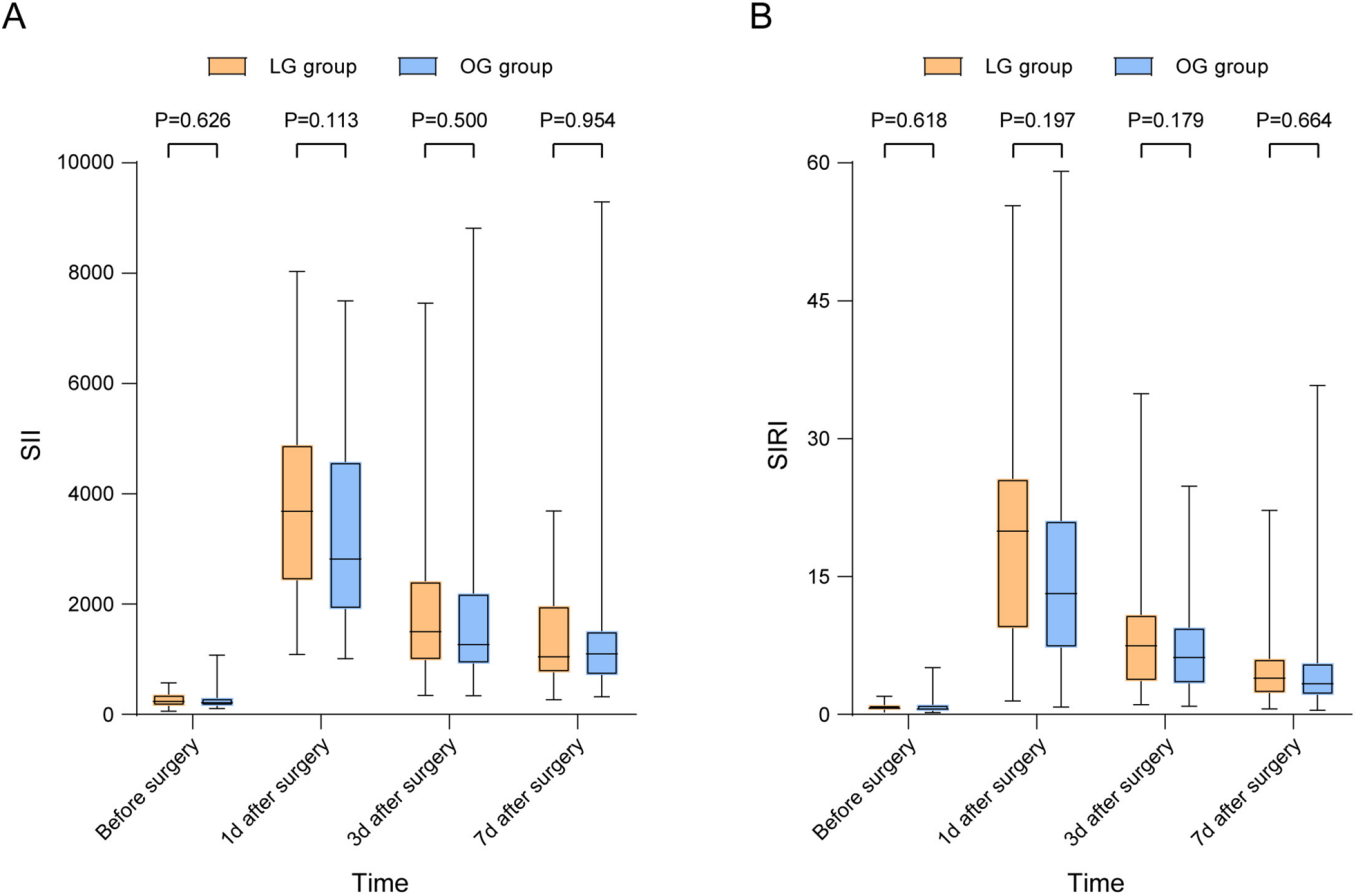
Perioperative immune-inflammation status of the LG and OG groups. The SII was calculated as neutrophil count × platelet count/lymphocyte count. The SIRI was calculated as neutrophil count × monocyte count/lymphocyte count. LG, laparoscopic gastrectomy; OG, open gastrectomy; SII, systemic immune-inflammation index; SIRI, systemic inflammatory response index

In order to comprehensively figure out the influence of different surgical methods on LAGC patients following NC, we collected perioperative laboratory data to analyze the nutritional and immune-inflammation status of patients during the perioperative period. By calculating the reduction of red blood cells (RBCs) and Hb, we found that LG group had less reduced RBCs (*P* = 0.039) and Hb (*P* = 0.018) in 3 days after surgery (Fig. 2A–B), which confirmed that LG group had less blood loss than OG group during surgery. This may be attributed to the smaller incision size, better visualization of anatomical structures, and more precise hemostasis achieved by laparoscopic surgery. The reduced Alb was also calculated and found to be less in LG group both in 1 day (*P* = 0.029), 3 days (*P* = 0.015), and 7 days (*P* = 0.035) after surgery (Fig. 2D). PAB is a good indicator for short-term nutritional status due to its short half-life period [22, 23]. Our results showed that LG group had less reduced PAB in 3 days after surgery (*P* = 0.010) (Fig. 2E). All above results indicated that LG is less harmful to the nutritional status of LAGC patients after NC. The SII and SIRI are generally acknowledged indicators for the immune-inflammation status of patients [24, 25]. By calculating the SII and SIRI before surgery and 1 day, 3 days, and 7 days after surgery, we found no significant difference between the two groups (Fig. 3), which further proved the safety of LG for LAGC patients after NC.

### Oncological outcomes

**Fig. 4:**
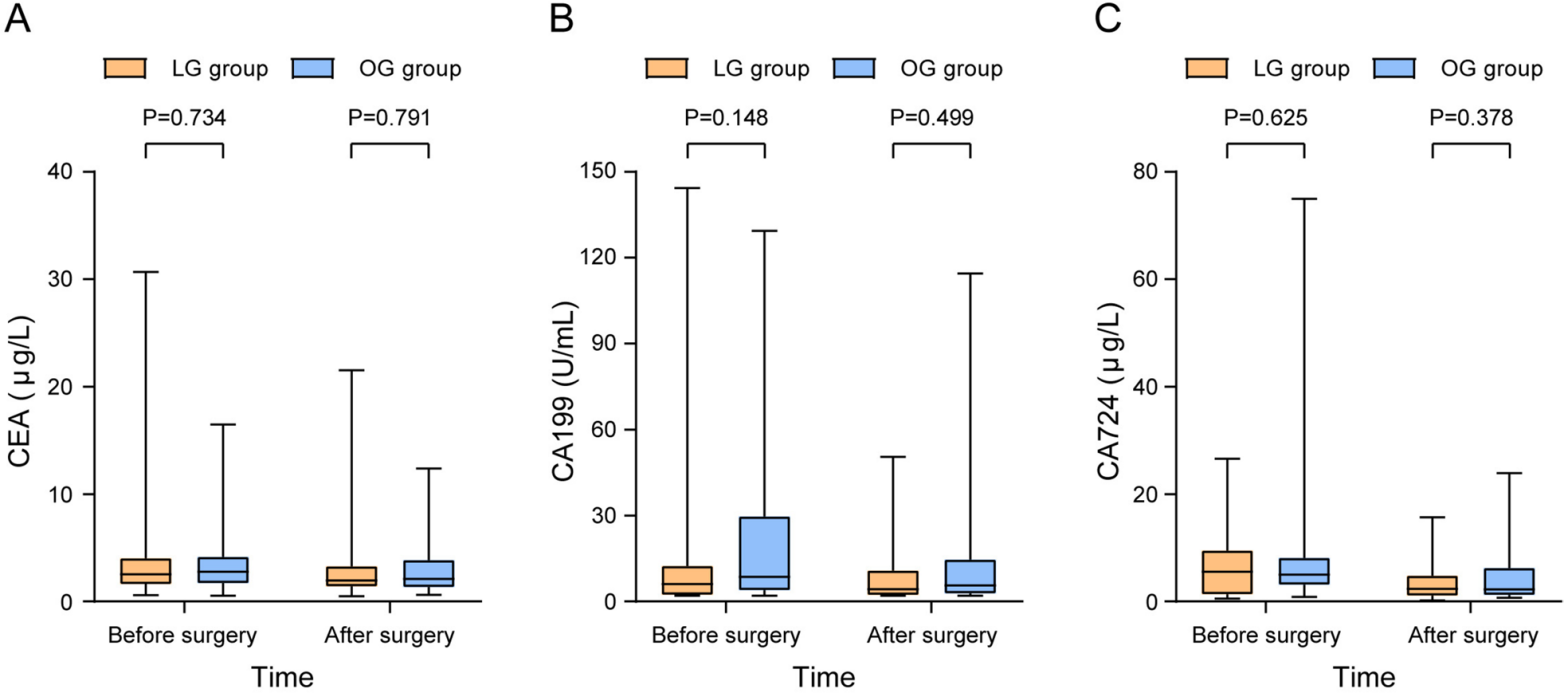
Tumor markers before and after surgery. The value referred to the laboratory results at 1 week before surgery and 1 month after surgery. LG, laparoscopic gastrectomy; OG, open gastrectomy

**Fig. 5:**
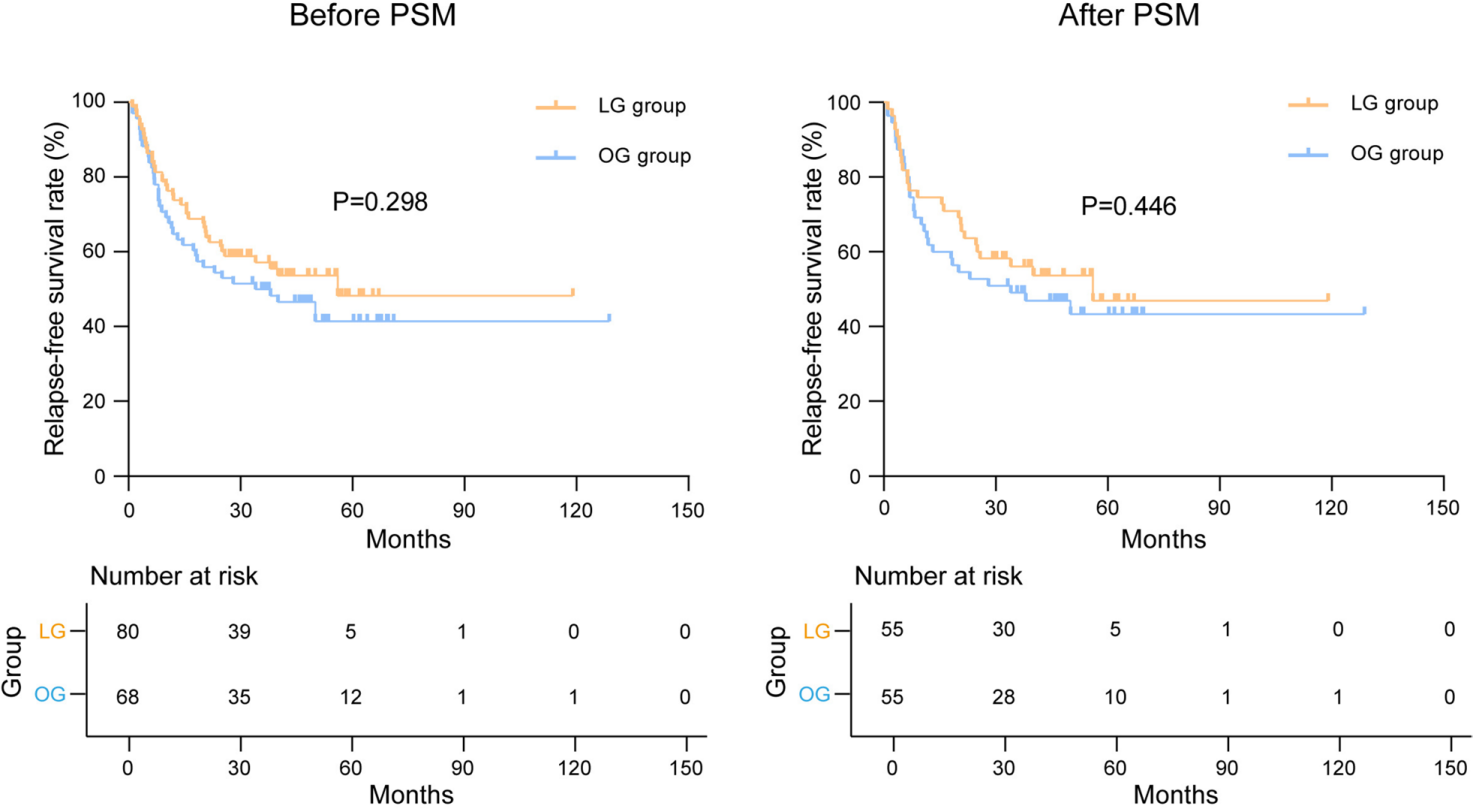
Kaplan–Meier analysis of relapse-free survival before and after PSM. PSM, propensity score matching; LG, laparoscopic gastrectomy; OG, open gastrectomy

**Fig. 6:**
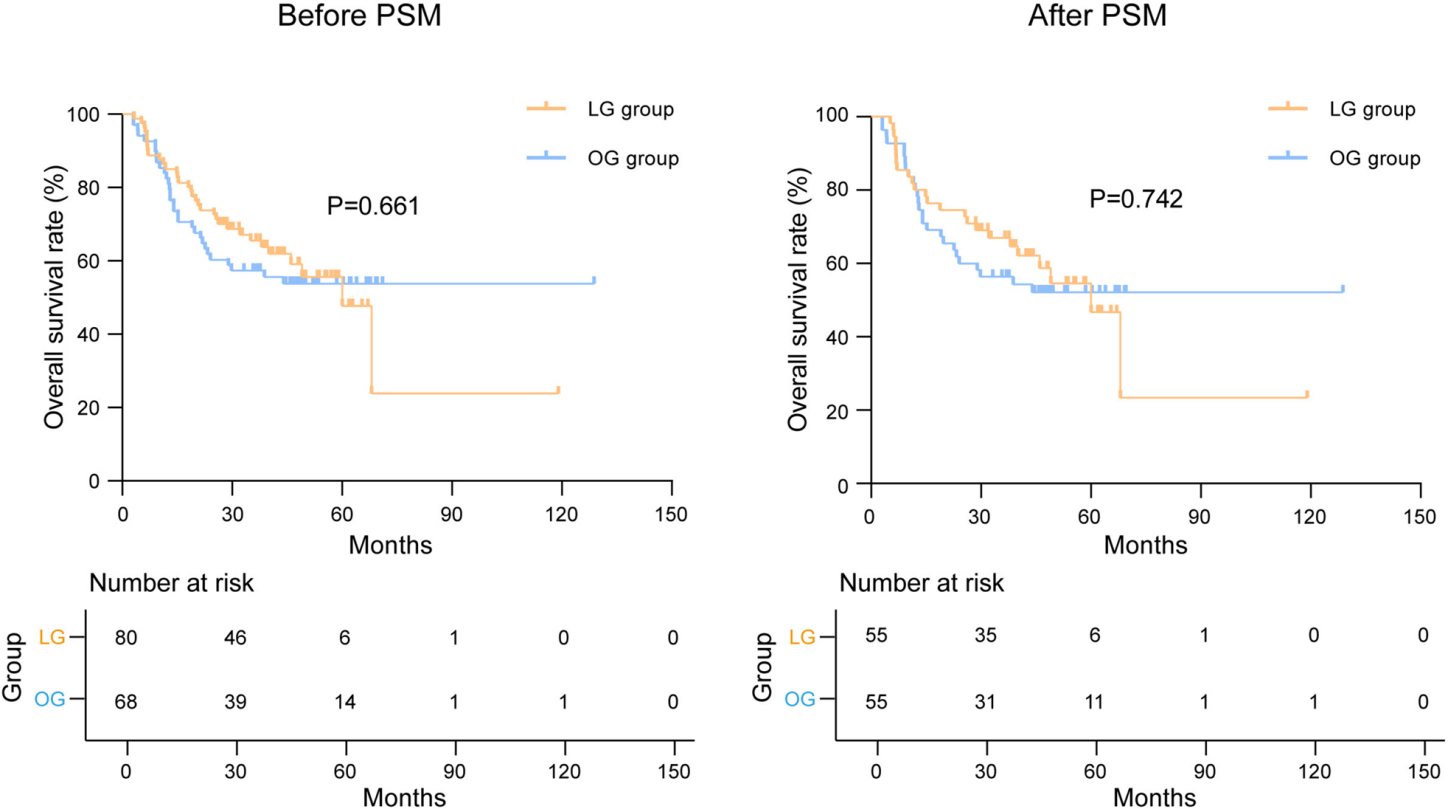
Kaplan–Meier analysis of overall survival before and after PSM. PSM, propensity score matching; LG, laparoscopic gastrectomy; OG, open gastrectomy

To evaluate the short-term oncological outcomes of different surgical methods, the data of tumor markers at a week before surgery and 1 month after surgery were collected. The results showed no significant difference between the two groups (Fig. 4). Regarding the long-term oncological outcomes, we recorded and analyzed the recurrence and survival status of patients after a median follow-up of 48.0 (3.0–128.8) months. The overall recurrence within 3 years occurred in 24 (43.6%) and 28 (50.9%) patients in the LG and OG groups, respectively (*P* = 0.445). And there were no significant differences in local recurrence (*P* = 0.768) and metastasis (*P* = 0.550) between the two groups. The 5-year RFS and 5-year OS rates in the LG group were 46.9% and 46.7%, respectively, and the corresponding rates in the OG group were 43.3% and 52.1%, respectively. No significant difference was found in RFS (*P* = 0.446) or OS (*P* = 0.742) (Table 5). The Kaplan–Meier analysis showed no significant difference in RFS and OS both before and after PSM (Figs. 5 and 6).

**Table 5:** Postoperative recurrence and survival in the LG and OG groups after PSM

Characteristics	LG group (*n* = 55)	OG group (*n* = 55)	*Χ*^2^	*p*-value
Overall recurrence^a^, no. (%)	24 (43.6)	28 (50.9)	0.584	0.445
Local recurrence^a^, no. (%)	6 (10.9)	7 (12.7)	0.087	0.768
Metastasis^a^, no. (%)	18 (32.7)	21 (38.2)	0.358	0.550
Liver	8 (14.5)	8 (14.5)		
Peritoneum	2 (3.6)	5 (9.1)		
Liver and peritoneum	3 (5.5)	1 (1.8)		
Lung	0 (0.0)	2 (3.6)		
Pancreas	1 (1.8)	2 (3.6)		
Ovary	2 (3.6)	1 (1.8)		
Bone	1 (1.8)	2 (3.6)		
Kidney	1 (1.8)	0 (0.0)		
RFS (%)			0.580	0.446
1-year RFS	74.5	61.8		
3-year RFS	56.0	49.0		
5-year RFS	46.9	43.3		
OS (%)			0.108	0.742
1-year OS	80.0	81.8		
3-year OS	67.0	56.4		
5-year OS	46.7	52.1		

*PSM* propensity score matching, *LG* laparoscopic gastrectomy, *OG* open gastrectomy, *RFS* relapse-free survival, *OS* overall survival

^a^Refer to recurrence within 3 years

### Risk factors of RFS and OS

Univariate Cox regression analysis showed that surgical procedures were not relevant to RFS (*P* = 0.448) or OS (*P* = 0.742). The risk factors related to RFS were tumor size ≥ 4 cm, lymph-vascular invasion, nerve invasion, R1 resection, clinical response of RECIST being SD or PD, ypTNM stages III or IV, and the absence of postoperative adjuvant chemotherapy. The risk factors related to OS were tumor size ≥ 4 cm, lymph-vascular invasion, nerve invasion, R1 resection, clinical response of RECIST being SD or PD, ypTNM stages III or IV, and the absence of postoperative adjuvant chemotherapy (Table 6). Multivariate Cox regression analysis showed that tumor size ≥ 4 cm (*P* = 0.021) and the absence of postoperative adjuvant chemotherapy (*P* = 0.012) were independent risk factors of OS (Table 7).

**Table 6:** Univariate analysis of risk factors for RFS and OS

Variables	RFS			OS		
	*HR*	95% *CI*	*p*-value	*HR*	95% *CI*	*p*-value
Age (≥ 60 *vs.* < 60 years)	1.192	0.706–2.014	0.511	1.142	0.654–1.993	0.641
Sex (female *vs.* male)	1.681	0.902–3.131	0.102	1.694	0.884–3.246	0.112
CCI^a^ (≥ 2 *vs.* < 2)	1.293	0.585–2.858	0.526	0.991	0.392–2.503	0.984
cTNM stage^b^ (III *vs.* II)	1.149	0.520–2.538	0.731	0.974	0.437–2.167	0.948
Surgical procedure (laparoscopy *vs.* open)	0.816	0.482–1.380	0.448	0.911	0.522–1.589	0.742
Number of lymph node dissection (≤ 22 *vs.* < 22)	0.951	0.563–1.606	0.850	1.060	0.606–1.855	0.838
Tumor size^c^ (≥ 4 *vs.* < 4 cm)	2.621	1.491–4.606	0.001	3.792	2.001–7.186	< 0.001
Histological type (undifferentiated *vs.* differentiated)	1.695	0.911–3.153	0.096	1.801	0.922–3.520	0.085
Lymph-vascular invasion (yes *vs.* no)	3.175	1.850–5.447	< 0.001	3.234	1.829–5.718	< 0.001
Nerve invasion (yes *vs.* no)	1.973	1.160–3.356	0.012	1.814	1.037–3.174	0.037
Margin of resection (R1 *vs.* R0)	3.457	1.718–6.957	0.001	3.201	1.542–6.642	0.002
Postoperative complications (yes *vs.* no)	1.351	0.738–2.476	0.330	1.279	0.668–2.448	0.458
Clinical response of RECIST^d^ (PD or SD *vs.* PR or CR)	7.755	3.761–15.991	< 0.001	8.506	3.787–19.104	< 0.001
Tumor regression grade^e^ (2 or 3 *vs.* 1 or 0)	2.059	0.881–4.810	0.095	2.300	0.908–5.829	0.079
ypTNM stage^b^ (III or IV *vs.* PCR or I or II)	6.841	3.497–13.381	< 0.001	7.565	3.519–16.264	< 0.001
Postoperative adjuvant chemotherapy (no *vs.* yes)	2.080	1.132–3.825	0.018	2.350	1.259–4.384	0.007

*RFS* relapse-free survival, *OS* overall survival, *CCI* Charlson Comorbidity Index, *RECIST* Response Evaluation Criteria in Solid Tumors, *CR* complete response, *PR* partial response, *SD* stable disease, *PD* progressed disease, *PCR* pathological complete response

^a^According to the Charlson Comorbidity Index

^b^According to the 8th edition of the *American Joint Committee on Cancer Cancer Staging Manual*

^c^Refer to the long diameter of tumor after neoadjuvant chemotherapy

^d^According to the Response Evaluation Criteria in Solid Tumors (RECIST version 1.1)

^e^According to the AJCC standard

**Table 7:** Multivariate analysis of risk factors for RFS and OS

Variables	RFS			OS		
	*HR*	95% *CI*	*p*-value	*HR*	95% *CI*	*p*-value
Tumor size^a^ (≥ 4 *vs.* < 4 cm)	1.448	0.788–2.659	0.233	2.230	1.131–4.400	0.021
Histological type (undifferentiated *vs.* differentiated)	1.026	0.537–1.958	0.939	1.051	0.516–2.140	0.890
Lymph-vascular invasion (yes *vs.* no)	1.328	0.737–2.392	0.346	1.414	0.751–2.663	0.283
Nerve invasion (yes *vs.* no)	0.989	0.497–1.971	0.976	0.956	0.459–1.993	0.905
Margin of resection (R1 *vs.* R0)	1.300	0.554–3.046	0.547	0.928	0.382–2.253	0.869
Clinical response of RECIST^b^ (PD or SD *vs.* PR or CR)	3.273	0.913–11.730	0.069	3.125	0.810–12.059	0.098
Tumor regression grade^c^ (2 or 3 *vs.* 1 or 0)	0.795	0.278–2.271	0.668	1.027	0.316–3.325	0.965
ypTNM stage^d^ (3 or 4 *vs.* PCR or 1 or 2)	2.386	0.805–7.068	0.117	2.509	0.787–8.000	0.120
Postoperative adjuvant chemotherapy (no *vs.* yes)	2.204	0.983–4.941	0.055	2.903	1.261–6.684	0.012

*RFS* relapse-free survival, *OS* overall survival, *RECIST* Response Evaluation Criteria in Solid Tumors, *CR* complete response, *PR* partial response, *SD* stable disease, *PD* progressed disease, *PCR* pathological complete response

^a^Referred to the long diameter of tumor after neoadjuvant chemotherapy

^b^According to the Response Evaluation Criteria in Solid Tumors (RECIST version 1.1)

^c^According to the AJCC standard

^d^According to the 8th edition of the *American Joint Committee on Cancer Cancer Staging Manual*

## Discussion

Surgery is the first-line treatment for LAGC. The principle of surgery is the complete resection of tumor and adequate lymphadenectomy. The JCOG0912 trial [26], KLASS01 trial [27], and CLASS02 trial [28] have proved the safety and feasibility of LG for early gastric cancer. Similarly, the CLASS01 trial [3–5] and KLASS02 trial [6, 7] observed that laparoscopic distal gastrectomy is safe and feasible for LAGC. However, the participants included in the above studies were gastric cancer patients who did not receive NC. Most previous retrospective studies on the outcomes of laparoscopic surgery for LAGC after NC showed significant differences in baseline data [29–31]. And seldom have studies compared the nutritional status, immune-inflammation conditions, and tumor markers between the two different surgical methods. To the best of our knowledge, this is the first study to comprehensively evaluate the short- and long-term outcomes of laparoscopic surgery for LAGC after NC with clinicopathological, prognostic, and laboratory data after balancing the differences between the two groups with PSM. Our results confirmed the safety and efficacy of LG in LAGC patients following NC. What’s more, we found that LG had faster gastrointestinal recovery, better postoperative nutritional status than OG, which allows LG to have extensive application in the background of enhanced recovery after surgery (ERAS).

Local tumors are prone to tissue edema, exudation, and fibrosis after NC, which may affect the identification of anatomic spaces and increase the difficulty of surgery as well as the risk of postoperative complications [32]. However, the FNCLCC FFCD trial showed that the occurrence rate of complications in surgery following NC group was 25.7% (28/109), which was not significantly different from 19.1% (21/110) in the surgery-only group for resectable gastroesophageal adenocarcinoma [10]. Previous studies showed that the occurrence rate of postoperative complications in LAGC patients who did not receive NC was 14.1–38.0% [3, 6, 33–35]. The overall incidence of postoperative complications in our study was 20.9%, which indicated that NC did not obviously increase the incidence of postoperative complications.

Laparoscopic surgery involves a smaller incision and can effectively avoid tissue pull injury caused by open surgery. In addition, the visual magnification of laparoscopy allows a clearer surgical field and improves visualization of anatomical layers, resulting in less intraoperative damage to the surrounding tissues. Our study showed that patients in LG group had less blood loss during surgery and had less reduced Alb and PAB after surgery, which indicated a better postoperative nutritional status. This could be due to less damage to the gastrointestinal tract, which allows faster recovery of gastrointestinal function and earlier time to first liquid diet. Related study reported that the faster recovery of postoperative nutritional status in laparoscopic surgery patients may also be associated with less postoperative pain, early-stage physical exercise, and smaller stress response [36]. Meanwhile, Alb and PAB were reported to be closely related to the prognosis of cancer patients [37–40], which indicated that LG might have potential benefit of improving prognosis. Above all, these advantages allow LG to have promising application in the era of minimally invasive surgery and ERAS [41, 42].

The number of LND is closely related to postoperative pathological stage and prognosis evaluation of gastric cancer. Some lymph nodes retreat after NC, which may lead to a reduced number of LND. Therefore, a large bias may arise when predicting prognosis using the ypTNM staging system. The lymph node ratio (LNR) has been proved to be a more stable and accurate prognostic indicator than N stage in previous studies [43], and it is usually unaffected by the number of LND in predicting the prognosis of gastric cancer patients. Therefore, LNR is a promising prognostic indicator to replace the ypTNM staging system for LAGC after NC [44, 45]. In addition, several large RCTs confirmed that the number of LND by laparoscopic surgery was similar to that of open surgery for LAGC [3, 6, 46]. However, it is unknown whether LG can achieve the same number of LND as that of OG for LAGC after NC because of the fact that tissue exudation and edema caused by NC may affect the surgical process. Our results showed that the two surgical methods have similar number of LND, which is consistent with the results of Li et al. [12] and Fujisaki et al. [47]. Therefore, LG for LAGC patients after NC is safe and feasible in terms of lymphadenectomy.

Long-term follow-up findings in CLASS-01 [5] and KLASS-02 trials [7] showed that LG was not inferior to OG in the treatment of LAGC. With regard to the oncological outcomes of LG for LAGC after administration of NC, our study indicated that LG was comparable to those of OG both in the efficacy of decreasing tumor markers and long-term RFS and OS, which is consistent with previous literature reports [30, 31, 47]. Moreover, it is unclear whether postoperative adjuvant chemotherapy will further improve the prognosis of LAGC after NC. Our results showed that the absence of postoperative adjuvant chemotherapy was an independent risk factor to the OS of LAGC patients after NC. Therefore, it is necessary for LAGC patients following NC to receive postoperative adjuvant chemotherapy to achieve the best survival benefit as long as they are able to tolerate adverse effects.

Above all, this study made a comprehensive evaluation and proved the non-inferiority of LG to OG for LAGC patients following NC. However, as this was a retrospective study of single center, it had limited sample size and could not avoid the inherent bias of the retrospective study design. Besides, LG is a novel surgical method emerging in recent years. The widespread application of LG is later than traditional OG, especially for LAGC patients after NC. Therefore, the follow-up time of LG was shorter than that of OG, which may affect the long-term survival results between the two groups. However, the median follow-up time in both groups was greater than 36 months, which provides preliminary evidence for the oncologic safety of LG. Certainly, we will keep on following up and look forward to conducting multicenter prospective RCT to derive more rigorous and accurate conclusions.

## Conclusions

In conclusion, LG has a shorter length of incision, faster gastrointestinal recovery, better postoperative nutritional status, and comparable oncological outcomes than OG and can serve as an alternative surgical method for LAGC patients after NC.

## Data Availability

The datasets used and/or analyzed during the current study are available from the corresponding author on reasonable request.

## Abbreviations

LAGC: Locally advanced gastric cancer
NC: Neoadjuvant chemotherapy
RCT: Randomized controlled trial
LG: Laparoscopic gastrectomy
OG: Open gastrectomy
PSM: Propensity score matching
CCI: Charlson Comorbidity Index
ASA: American Society of Anesthesiologists
AJCC: American Joint Committee on Cancer
RECIST: Response Evaluation Criteria in Solid Tumors
CT: Computed tomography
LND: Lymph node dissection
RFS: Relapse-free survival
OS: Overall survival
LNR: Lymph node ratio
SII: Systemic immune-inflammation index
SIRI: Systemic inflammatory response index
CR: Complete response
PR: Partial response
SD: Stable disease
PD: Progressed disease
